# Handling intercurrent events and missing data in non-inferiority trials using the estimand framework: A tuberculosis case study

**DOI:** 10.1177/17407745231176773

**Published:** 2023-06-05

**Authors:** Sunita Rehal, Suzie Cro, Patrick PJ Phillips, Katherine Fielding, James R Carpenter

**Affiliations:** 1 GlaxoSmithKline, Middlesex, UK; 2 Imperial Clinical Trials Unit, School of Public Health, Imperial College London, London, UK; 3 UCSF Center for Tuberculosis, University of California San Francisco, San Francisco, CA, USA; 4 London School of Hygiene and Tropical Medicine, London, UK; 5 Medical Research Council Clinical Trials Unit, University College London, London, UK

**Keywords:** Binary outcome, estimands, intercurrent events, missing data, multiple imputation, non-inferiority, reference-based sensitivity analysis, sensitivity analyses

## Abstract

**Introduction:**

The ICH E9 addendum outlining the estimand framework for clinical trials was published in 2019 but provides limited guidance around how to handle intercurrent events for non-inferiority studies. Once an estimand is defined, it is also unclear how to deal with missing values using principled analyses for non-inferiority studies.

**Methods:**

Using a tuberculosis clinical trial as a case study, we propose a primary estimand, and an additional estimand suitable for non-inferiority studies. For estimation, multiple imputation methods that align with the estimands for both primary and sensitivity analysis are proposed. We demonstrate estimation methods using the twofold fully conditional specification multiple imputation algorithm and then extend and use reference-based multiple imputation for a binary outcome to target the relevant estimands, proposing sensitivity analyses under each. We compare the results from using these multiple imputation methods with those from the original study.

**Results:**

Consistent with the ICH E9 addendum, estimands can be constructed for a non-inferiority trial which improves on the per-protocol/intention-to-treat-type analysis population previously advocated, involving respectively a hypothetical or treatment policy strategy to handle relevant intercurrent events. Results from using the ‘twofold’ multiple imputation approach to estimate the primary hypothetical estimand, and using reference-based methods for an additional treatment policy estimand, including sensitivity analyses to handle the missing data, were consistent with the original study’s reported per-protocol and intention-to-treat analysis in failing to demonstrate non-inferiority.

**Conclusions:**

Using carefully constructed estimands and appropriate primary and sensitivity estimators, using all the information available, results in a more principled and statistically rigorous approach to analysis. Doing so provides an accurate interpretation of the estimand.

## Introduction

The ICH E9 addendum^1^ advocates increased clarity around the primary objective in clinical trials, by requesting a precise definition of the targeted treatment effect, which is referred to as the estimand. An estimand consists of five components: the target population, outcome variable, treatment condition, population-level summary measure and handling of intercurrent (i.e. post-baseline) events. An intercurrent event (IE) is an event which occurs after treatment initiation that affects either the interpretation or the existence of the measurements associated with the clinical question of interest.^1^ The addendum acknowledges that the estimand for a non-inferiority study design may differ markedly from those which inform a superiority study. This is because such trials are not ‘conservative’ in nature, meaning that IEs such as use of rescue medication may bias the result towards a conclusion of non-inferiority. However, no further guidance on estimand construction for non-inferiority trials is provided.

The addendum proposes various strategies for handling IEs.^1^ We focus on the *hypothetical strategy* which seeks to estimate the treatment effect had the IE not occurred (e.g. if the IE was use of rescue medication the treatment effect seeks to estimate what would have happened if all patients took treatment per-protocol (PP)) in the absence of rescue medication, and the *treatment policy strategy* which seeks to estimate the treatment effect using all outcomes regardless of the IE (e.g. targets the treatment effect regardless of use of rescue medication). Alternative strategies for handling IEs include composite, while-on-treatment and principal stratification.^1^

Prior to the estimand framework, IEs such as use of rescue medication in non-inferiority trials were dealt with using a PP analysis and intention-to-treat analysis with any differences in conclusions between the two investigated.^2–5^ This was due to the intention-to-treat analysis being anti-conservative in non-inferiority trials as the investigated treatment may look similar to the control, indicating a treatment effect of no difference.^6^ The intention-to-treat analysis typically involves analysing all patients as randomised regardless of subsequent behaviour. This closely aligns with a treatment policy strategy where outcomes following the IEs are included in the analysis. For the PP analysis, participants experiencing IEs are entirely excluded; it is not as clear-cut what strategy this corresponds to without further details of the analyst’s assumptions. Further, the addendum states that it may not be possible to construct a relevant estimand to which analysis of the PP set is aligned.^1^ Clearly under the new framework only specifying the participants included in the analysis sets (the analysis populations) is no longer sufficient, and what one wishes to estimate including the desired strategy for handling the IEs must be clarified instead of simply excluded. Further, if data after an IE are missing, but would have been relevant for the estimand of interest (e.g. when using a treatment policy strategy) additional considerations for estimation arise. In this scenario, aligned with the estimand, a principled approach we explore to handle missing data is to use multiple imputation^7^ to impute the missing data after the IE using the information observed up to the point of the IE.^8–10^

As in superiority trials, the analysis of a non-inferiority trial may additionally be complicated by missing outcome data, for example due to missed patient visits. This is because any analysis will then involve untestable assumptions about the distribution of these data. A key aspect of the estimand framework is that missing data is a problem for the estimator not the estimand. Therefore, missing data is not to be viewed as an IE but there may also be missing data as a *consequence* of an IE.

When missing outcome data arises, it is acknowledged^11–13^ that analysis should not only consist of a primary analysis under the most plausible missing data assumption but also include sensitivity analysis, under alternative contextually plausible assumptions concerning the distribution of the unobserved data.^14^ In practice, sensitivity analyses in non-inferiority studies are often simplistic, and make extreme assumptions – for example best/worst case scenarios.^15,16^ Given the expense of clinical trials and the difficulty of recruiting patients to them, there is a need for improved handling of missing data for both primary and sensitivity analyses. This article proposes methods for handling missing data in non-inferiority trials,^15^ and focuses on a binary composite outcome. First, we introduce our motivating example, REMoxTB: a phase III non-inferiority tuberculosis study with longitudinal binary outcome data. We then aim to (1) define and show how relevant estimands can be constructed for a non-inferiority study, (2) demonstrate estimators for targeting the relevant estimands using multiple imputation methods and (3) extend and use the recent *reference-based* sensitivity analyses methodology^17^ for a binary outcome.

## Methods

### Case study: 4-month moxifloxacin-based regimens for drug-sensitive tuberculosis (REMoxTB)

REMoxTB^18^ was a 3-arm double-blind, placebo-controlled non-inferiority trial, randomising 1931 patients 1:1:1 to either the standard treatment regimen or two shorter treatment combinations, in patients with newly diagnosed pulmonary tuberculosis.

Patients were requested to attend clinic every week for the first 8 weeks, then at 12, 17, 22, 26, 38, 52, 65 and 78 weeks. At each visit, at least one sputum sample was taken and sent to a laboratory to test for the presence (i.e. a positive result)/absence (i.e. a negative result) of mycobacteria tuberculosis. Contaminated results were considered as missing data. The primary outcome was defined as a binary composite of treatment failure or relapse over the course of the 78-week follow-up. Each patient would be classed as a ‘success’ or ‘failure’ at the end of follow-up (78 weeks) as described below. We note that to avoid confusion with the composite strategy when defining the estimand below, we will no longer refer to this endpoint as being a composite.

### Definition of primary outcome

#### Favourable outcome

**Figure 1.**
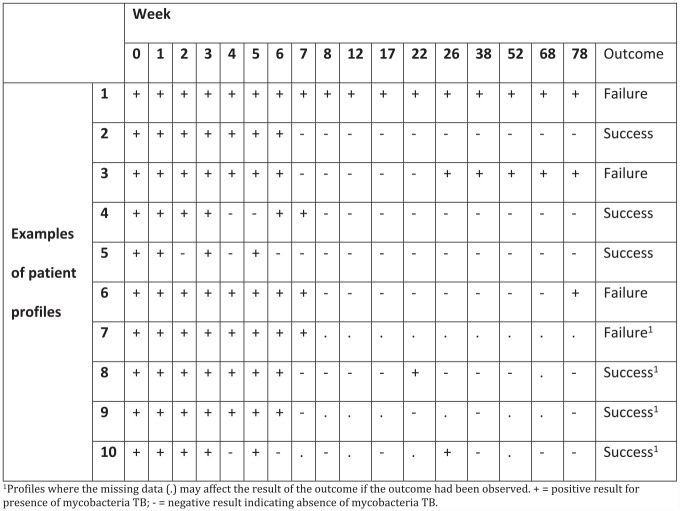
Examples of test results from patient profiles complicated by missing data. ^1^Profiles where the missing data (.) may affect the result of the outcome if the outcome had been observed. + = positive result for presence of mycobacteria TB; − = negative result indicating absence of mycobacteria TB.

- A patient was deemed to have a favourable outcome if they achieved stable negative culture conversion.
- Stable negative culture conversion: Occurs when a patient has at least two consecutive negative culture results at two different scheduled visits *without* an intervening positive culture result over 78 weeks (e.g. profile 2 in Figure 1).

#### Unfavourable outcome

- A patient is considered to have an unfavourable outcome in the case of relapse or treatment failure.
- Relapse: A patient who has two consecutive positive cultures at different visits, without an intervening negative culture, after achieving stable negative culture conversion (e.g. profile 3 in Figure 1).
- Treatment failure: A patient who either never achieves a favourable outcome (e.g. profile 1 in Figure 1), or whose last observed result was positive (e.g. profile 6 in Figure 1).

Note that almost all patients achieve negative culture status in the first 4 to 6 months of the intensive treatment phase. The key question is whether this is sustained throughout the follow-up for the new, shorter, treatment regimens. This definition is complicated by the fact that missing data could affect the classification of whether a patient is considered to meet the endpoint (see Figure 1 for examples of patient profiles).

In the original REMoxTB study, any patients with negative test results (confirmed not to have tuberculosis) post-randomisation only after more accurate testing were excluded due to the absence of the disease at baseline (not eligible). This resulted in 146 patients being excluded.^18^ These patients remain excluded from the analyses explored here. Excluding such patients will not introduce bias since their exclusion is not influenced by post-randomisation events.^19^

### Defining the estimands relevant for a non-inferiority study – application to REMoxTB

We first focus on *IEs* for REMoxTB and define two estimands (primary and additional) suitable for a non-inferiority trial by considering how the original study defined the analysis population according to modified intention-to-treat (mITT) and PP definitions.^18^

The exclusions which defined these populations form the majority of the IEs. Table 1 summarises the proportion of patients in each treatment group having the IE and who were excluded from the mITT and PP analyses within the original trial analysis. Note, that having an unassessable sample or not reaching the end of the 6-month treatment phase are not IEs but are a missing data problem since the occurrence of these for a patient could affect whether they are considered to be favourable/unfavourable.

**Table 1.** Additional exclusions from the primary PP analysis and mITT analysis in the published REMoxTB analysis leading to missing data.

Reason	Control (*N* = 590)	Isoniazid (*N* = 609)	Ethambutol (*N* = 586)	Total (*N* = 1785)
Exclusions from both published mITT and PP analysis				
Exogenous reinfection during follow-up^1^	10 (2%)	8 (1%)	13 (2%)	31 (2%)
Not assessable at 78 weeks, but culture negative when last seen	24 (4%)	24 (4%)	16 (3%)	64 (4%)
Withdrawal from trial medications due to pregnancy^1^	1 (0.2%)	4 (0.7%)	0	5 (0.3%)
Exogenous reinfection during treatment^1^	0	5 (0.8%)	6 (1%)	11 (0.6%)
Additional exclusions from published PP analysis				
Change treatment for reasons other than treatment failure^1^	30 (5%)	42 (7%)	21 (4%)	93 (5%)
Patient did not reach the end of treatment phase (month 6)	13 (2%)	10 (2%)	6 (1%)	29 (2%)
Major protocol violation before unfavourable outcome^1^	2 (0.3%)	0	0	2 (0.1%)
Patient did not receive adequate active drug^1^	0	2 (0.3%)	0	2 (0.1%)
Total	80 (14%)	95 (16%)	62 (11%)	237 (21%)

PP: per-protocol.

1 Considered as an intercurrent event.

Instead of excluding patients who have an IE (and those missing outcome data) as done in the original study analysis (Table 1), each IE can be appropriately handled so that the treatment effect being targeted can be understood. IE strategies can be used alone or in combination to address multiple IEs.^20^ We assume that the clinical question of primary interest is to assess the treatment effect if it were taken as directed and IEs such as pregnancy and/or reinfection did not occur. Therefore, the targeted treatment effect to be estimated is a scenario in which we envisage these IEs did not occur: a hypothetical strategy.

Consistent with the estimand framework, the primary estimand we infer is defined in Table 2. As an additional estimand to the primary estimand, we propose IEs are handled using a treatment policy strategy to determine the treatment effect including all outcomes regardless of the IEs occurring.

**Table 2.** Definition of all five attributes used to construct the primary estimand for the REMoxTB study.

Primary estimand	
Population	Adult patients with newly diagnosed mycobacterium TB, not resistant to rifampicin or fluoroquinolones (as defined by trial inclusion/exclusion criteria).
Variable	An unfavourable outcome (defined as relapse or treatment failures) at week 78 as derived from the analysis of sputum samples.
Treatment	•Rifampicin (R), isoniazid (H), pyrazinamide (Z) and ethambutol (E) for 8 weeks followed by 18 weeks of rifampicin and isoniazid (control group) versus with a combination of R, H, Z and moxifloxacin (M) for 17 weeks followed by 9 weeks of placebo (isoniazid group) or •Rifampicin (R), isoniazid (H), pyrazinamide (Z) and ethambutol (E) for 8 weeks followed by 18 weeks of HR (control group) versus a combination of R, M, Z and E for 17 weeks followed by 9 weeks of placebo (ethambutol group).
Population-summary measure	A difference in proportions for each treatment comparison.
Intercurrent event and strategy	•Exogenous reinfection during follow-up. •Withdrawal from trial medications due to pregnancy. •Exogenous reinfection during treatment. •Change treatment for reasons other than treatment failure. •Major protocol violation before unfavourable outcome. •Patient did not receive adequate active drug.
All IEs are to be dealt with using a hypothetical strategy to determine the treatment effect in the absence of the IEs defined in Table 2.	

IE, intercurrent event; TB, tuberculosis.

### Estimators

To estimate the primary estimand, we propose to use the observed data (positive or negative presence of mycobacteria tuberculosis) up to the point the IE happens. Once the IE occurs, data are no longer collected and are imputed/predicted after this point, as aligned with the hypothetical strategy. For the primary statistical analysis, missing responses that occur after the IE are assumed to be missing at random. This means missing observations from those who experienced the IE are assumed to be similar to the responses observed for those who did not experience the IE. Multiple imputation methods (technical details for REMoxTB described below) can be used to appropriately account for the uncertainty in this missing data under missing at random.^7^

Patients with missing data who did not experience an IE (i.e. those in REMoxTB that were not assessable at 78 weeks, but culture negative when last seen or patients who did not reach the end of treatment phase (month 6)) will have missing responses imputed under missing at random. In addition, since intermittent missing data may affect the result of the outcome had the response been observed (Figure 1), intermittent missing responses will be imputed under on-treatment missing at random.

Sensitivity analyses can be performed for the primary estimand using a missing not at random assumption to explore the robustness of inferences to departures from missing at random under missing not at random. For estimation, missing not at random controlled multiple imputation methods can be employed which combine pattern-mixture modelling with multiple imputation and reference-based multiple imputation.^21,22^ To demonstrate sensitivity analysis and the applicability of missing not at random multiple imputation analyses within non-inferiority studies, we explore the last mean carried forwards approach. This approach assumes patients continued on their randomised arm (appropriate for the hypothetical strategy) but that their outcomes (missing post-IE) stay at the mean level for their randomised arm at their last observed time point. Specifically, the mean profile for patients is assumed to follow their randomised arms’ estimated mean profile until the IE or withdrawal, and then the estimated (marginal) treatment group mean at their last observed time point is carried forward and remains constant for each unobserved time point until the final follow-up visit. Then, conditional on the observed pre-IE/withdrawal data, the post-IE/withdrawal data are imputed from this distribution. It is a reference-based imputation method (technical details on how to implement for a binary outcome are described further below) where the reference is the patients’ randomised arm mean at their last observed time point.

To estimate the additional estimand, all data are considered regardless of the IE and missing data are imputed in line with the treatment policy strategy. That is, data are imputed to reflect what actually happened to the patients experiencing IEs rather than under hypothetical on-treatment behaviour. To address the missing data problem post-IE and other missing data, the jump-to-reference approach will be used under missing not at random.^21^ The jump-to-reference approach will assess the impact seen in practice where patients with missing data behave as though they are allocated the treatment reference (control) arm; a plausible assumption for this study given the nature of the IEs (e.g. study withdrawal and protocol violations) and disease. For sensitivity analysis, copy increments in reference and copy reference approaches will be used.^21^

#### Reference-based sensitivity analyses for a binary outcome via multiple imputation

For estimation under reference-based assumptions (including last mean carried forward), each patient’s conditional predictive (i.e. imputation) distribution is constructed under a specific assumption which is informed by other patients.^23^

This class of imputation methods was developed for a continuous outcome by Carpenter and Kenward^24^ based on ideas from Little and Yau^25^ and validated by Cro and her colleagues.^26–28^ Appendix B in the supplemental material gives more details of how this approach works for continuous data. We propose a novel extension to reference-based procedures for implementation with a longitudinal binary data. We propose to model the binary data as if it were continuous^21^ to carry out reference-based imputation. The joint model for each reference-based option is built under a multivariate normal distribution, without any modifications (Appendix B). The adaptive rounding algorithm proposed by Carpenter and Kenward,^24^ Horton et al.^29^ and Bernaards et al.^30^ is then used, where the binomial distribution is approximated to the normal distribution (Appendix C). The imputed observations are then back-transformed to binary observations (Appendix C). This transformation method has been shown to perform well under, and is preferred to other simplistic rounding approaches^30^ as it increases the variability for values that are imputed close to 0 or 1,^24^ an important component in the context of imputing for tuberculosis studies.

#### Additional analysis methods specific to the REMoxTB study data structure

For REMoxTB, we impute the binary variable measured at each follow-up visit (positive or negative for mycobacteria tuberculosis) over time. The primary outcome is a binary function of the whole history of the binary variable as described above. Once imputed, we then apply the study rules to determine the primary outcome, rather than solely imputing the outcome at the final visit.

**Figure 2.**
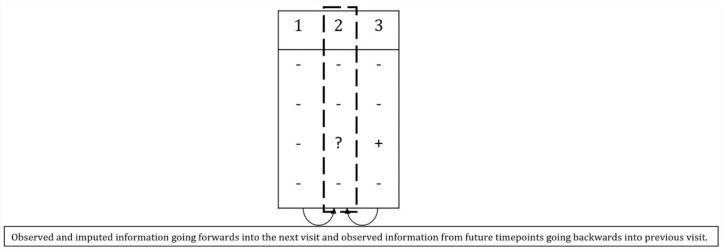
Concept of twofold fully conditional specification multiple imputation.

The REMoxTB study had 17 visits over a long period of time where a patient was tested for the absence or presence of tuberculosis. When the outcome is binary and the probabilities are close to 1 or 0, a flexible approach to impute the data is required to avoid computational issues due to collinearity and/or perfect prediction. To address this, the twofold multiple imputation approach proposed by Nevalainen et al.^31^ and validated by Welch et al.^32,33^ can be used assuming data are missing at random. Details of the approach are in Appendix A. Briefly, using multiple imputation,^7^ each outcome is imputed conditional on outcomes in a local visit window, rather than conditional on the full set of longitudinal outcomes. This window is then moved forward and backward through time a number of times, so that the whole data set is imputed. For example, Figure 2 shows the window at visit 2; missing outcome data at this visit are being imputed conditional on observed data from both visits 1 and 3. Data were imputed at each visit based on results on either side of that visit. Missing observations in the first and last visit can only be imputed based on the next visit and prior visit respectively.

**Figure 3.**
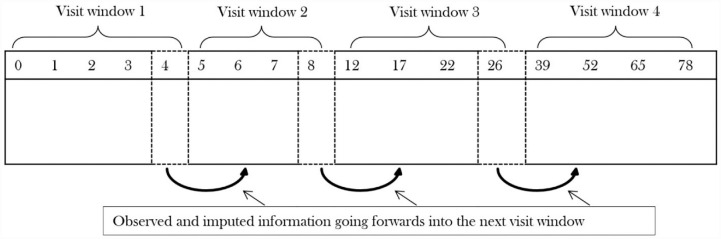
One ‘forwards pass’ approach to impute observations using reference-based multiple imputation.

Not unexpectedly, conducting reference-based sensitivity analyses via multiple imputation across all 17 visits proved difficult because – as mentioned above – of the large number of binary sequences that are mostly 1 or 0. To address this, the data were split into four clinically important visit windows. Data sets were imputed for the first visit window. Then, as shown in Figure 3, the last time from the first window was taken into window 2, which was then imputed once. Then the last time from window 2 was taken forward into window 3 which was then imputed, and so on. This procedure was repeated *K* times to create *K* imputed data sets. This is equivalent to one-forward pass of the twofold algorithm. We formed visit windows as weeks 0 to 4, 5 to 8, 12 to 26 and 39 to 78, and created 50 imputations.^34,35^

For each analysis, following multiple imputation, the difference in proportions between treatment (ethambutol group or isoniazid group) and control for the endpoint was calculated using a generalised linear model with an identity link as per the original study. Results across the multiply imputed data sets were combined for final inference using Rubin’s^7^ multiple imputation rules. The model included weight and centre as additional covariates and results were compared to a 6% non-inferiority margin using the upper bound of the two-sided 97.5% confidence interval (CI) (using Bonferroni correction), reflecting the original study.^18^ All analyses were conducted using Stata Version 14.1 (Appendix D). The estimates for the primary estimand (sensitivity analysis) will be compared with the result from the original PP analysis which, given our assumptions, is closely aligned with using the hypothetical strategy. The estimates for the additional estimand are compared with the result from the original mITT analysis as this is closely aligned with using a treatment policy strategy.

## Results

A total of 43% of patients (777/1785) had missing/contaminated results (either as a consequence of having an IE or general missing data) that could have directly influenced how they were classified at the end of the study: (256/590 (43%) on control; 268/609 (44%) on isoniazid and 253/586 (43%) on ethambutol. This is a non-trivial proportion of missing data that occur either as a direct consequence of the IE or are missing for reasons unknown.

### Primary estimand

**Figure 4.**
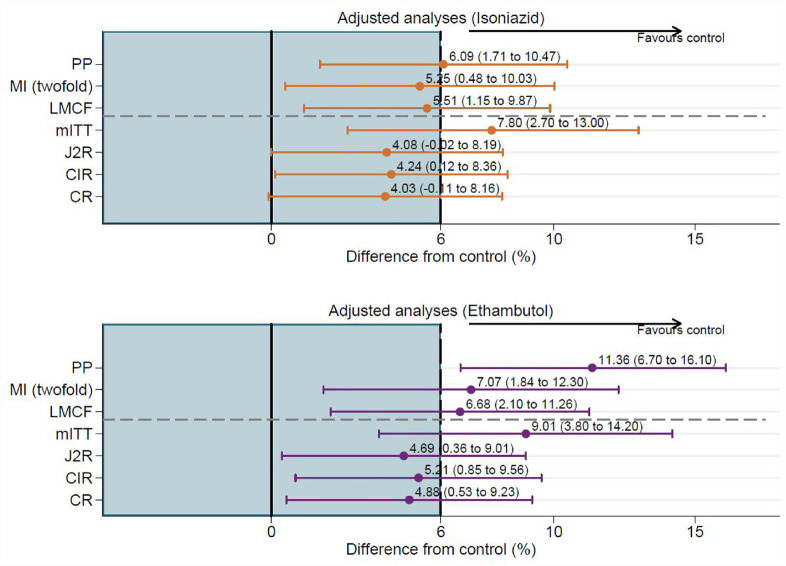
Difference in proportions and 97.5% CI using twofold fully conditional specification multiple imputation and reference-based sensitivity analyses. CI = confidence interval; PP = per-protocol analysis; MI (twofold) = twofold fully conditional specification multiple imputation analysis; LMCF = last mean carried forwards MI; mITT-modified intention-to-treat analysis; J2R = jump to reference MI; CIR-copy increments in reference MI; CR = copy reference MI.

The result for the primary estimand under our primary analysis assumption of MAR (obtained using twofold MI) is consistent with the trial’s reported primary PP analysis (see Figure 4). Both these and the original mITT analysis fail to demonstrate non-inferiority. For the PP analysis 80%, 76% and 70% of patients were considered to have reached culture-negative status by 78 weeks on the control, isoniazid and ethambutol arms, respectively. Twofold MI that included patient information that otherwise was discarded, on average, considerably increases the proportion of patients now considered to reach culture-negative status by 10% or more: 90% in the control arm, 85% in the isoniazid arm and 82% in the ethambutol arm.

### Sensitivity analysis for the primary estimand

For the last mean carried forwards sensitivity analysis, which assumes a patient’s mean probability of an unfavourable outcome does not change after loss to follow-up, is closer to our primary analysis (using the hypothetical strategy for all IEs) than to the reported primary PP analysis (most markedly for the ethambutol arm). The upper bound of the 97.5% CI was 9.87% for the isoniazid arm (5.51%; 97.5% CI: 1.15% to 9.87%) and 11.26% for the ethambutol arm (6.68%; 97.5% CI: 2.10% to 11.26%). Comparing these results to a 6% non-inferiority margin showed that – even under this assumption – the shorter 4-month treatment regimens failed to demonstrate non-inferiority favouring the control arm.

### Additional estimand and sensitivity analysis

The results for the additional estimand (using the treatment policy strategy for all IEs) under the assumption of jump-to-reference are not as extreme as those reported from the trial’s mITT analysis and the point estimates are slightly lower compared with the primary (missing at random) analysis (Figure 4). The results from the sensitivity analyses (copy increments in reference and copy reference) are very similar to the jump-to-reference analysis (the primary assumption for the additional estimand). Although all three reference-based approaches, which assume that patients with missing data in the isoniazid and ethambutol arms jump/revert/copy the control arm move towards non-inferiority, none of them meet the 6% margin (Figure 4); consistent with the trial’s reported mITT analysis.

## Discussion

In the context of non-inferiority trials in tuberculosis, we have constructed a primary estimand and an additional estimand and proposed principled primary and sensitivity estimators, which align with the relevant estimand while making full use of the observed information. We reconstructed the estimand for the REMoxTB study and inferred what strategies to use to handle the IEs given what the study estimated.

The conditions upon which patients were excluded in the original primary PP and mITT analyses for REMoxTB^18^ were defined as IEs and each was dealt with using a hypothetical strategy for the primary estimand and a treatment policy strategy for the additional estimand. A further complication to deal with was that intermittent missingness was ignored in the original analyses, despite the fact that the underlying (unseen) values could change the final outcome for patients. Instead, we imputed data that were missing after the IE using a hypothetical strategy, in addition to general missing data: (1) under missing at random in the primary analysis (in the absence of any IEs) with a sensitivity analysis using last mean carried forwards under missing not at random, then (2) in the additional estimand (using all outcomes regardless of the IEs) we made an assumption about what the post-IE data would have been under missing not at random (jump-to-reference) and used different sensitivity analyses (copy increments in reference and copy reference) aligned with this estimand. In each case, we used the imputed and observed data to derive the outcome for each patient.

Although we focussed throughout on a binary outcome, the multiple imputation methods discussed can be applied more broadly for non-inferiority trials across disease areas with other types of outcomes and trial designs.^21,36–38^

The results of REMoxTB using the twofold approach under missing at random (for the primary estimand) were consistent with the study results reported, where the alternative regimens failed to demonstrate non-inferiority. Results for the additional estimand and all sensitivity analyses under missing not at random were consistent across all options explored, providing greater confidence in the results provided by twofold multiple imputation. The point estimates and CIs from the study’s PP and mITT analysis are more extreme relative to results from the twofold multiple imputation and reference-based methods explored here. We note that the smaller estimates from using twofold multiple imputation could be due to the variability in the components of the primary estimand (using a hypothetical strategy) and the estimates of the CIs from the additional estimand and sensitivity analyses were also smaller, which could in part be a consequence of performing one ‘forwards’ pass of the twofold algorithm.

For REMoxTB,^18^ the handling of missing data was additionally complicated due to the long sequence of repeated binary outcomes observed at several time points (required to define the final result for each patient). Twofold multiple imputation, therefore, served as a more principled analysis under the missing at random assumption.^32,33^ The length of the visit window to impute the data at each time point was chosen one away from the current visit that needed imputing. A larger length may be chosen depending on how correlated patient outcomes are and whether the imputation model is computationally feasible. For fewer time points, standard missing at random multiple imputation can be used and other non-multiple imputation methods such as mixed-effects models could be considered for analysis under missing at random (aligned with the hypothetical strategy). As a first step, we recommend an attempt at using standard fully conditional specification multiple imputation under the missing at random assumption for long sequences of data.^31^ If that fails due to computational problems, we recommend using the twofold fully conditional specification^31^ multiple imputation outlined here.

To assess how robust the results were from using twofold multiple imputation, we applied reference-based sensitivity analyses assuming missing not at random to test for departures from the missing at random assumption.^21^ Other extensions have been considered for count^36^ and time-to-event^37^ outcomes. We extended this method for use with a binary outcome using the adaptive rounding algorithm,^30^ which worked well for this type of data. Reference-based methods, by nature, make the experimental drug look more similar to the reference/control arm. In clinical trials, where there is missing data, the ‘truth’ will always be unknown and therefore, plausible assumptions need to be made when using any analytical method. Therefore, in the context of non-inferiority studies, they should only be used when the underlying assumptions are plausible.

Other analyses, such as a tipping point analysis may be useful as a sensitivity analysis. We note that reference-based sensitivity analyses have the attraction of being ‘information anchored’.^17^ That is to say, the information lost due to missing data is held constant across the primary missing at random analysis and sensitivity analyses. This is an attractive property for regulators and trialists.

The additional estimand, which first employed a jump-to-reference assumption for missing data followed by sensitivity analyses under copy increments in reference and copy reference, showed some benefit on the alternative shorter regimens when assuming that patients follow the distribution of patients in the control arm when no longer observed. Since the shorter 17-week regimens performed poorly in comparison to the control, it is expected some benefit is shown when the intention-to-treat-type options are used (i.e. assuming a patients’ distribution follows that of the ‘better’ control treatment). Similarly, it is expected for patients to continue declining when assuming patients continue on the shorter treatment regimen as the treatment failed to demonstrate non-inferiority, as seen under the last mean carried forwards approach. The choice of a more ‘conservative’ analysis depends on where the estimate of the primary analysis lies relative to the margin. Therefore, we exercise caution in that the reference-based option(s) chosen to construct the joint multivariate normal distribution for non-inferiority studies can alter the severity of the statistical assumptions made and should not be chosen to intentionally demonstrate non-inferiority. We also highlight the important role sensitivity analyses have within the estimand framework for non-inferiority studies, so that decision-makers can be confident that the conclusions made from the chosen estimand are robust.

All IEs were dealt with using the same strategy to replicate what was done in the original trial (hypothetical for the primary estimand and treatment policy for the additional estimand). For new non-inferiority trials designed, the choice of the handling strategy for the primary estimand will depend on the clinical question of interest and therefore may differ depending on the study’s context. For tuberculosis studies, common IEs may need to be handled with different strategies than those proposed here depending on the stakeholder as proposed by Pham et al.^39^

An important aspect to consider for new non-inferiority studies is the extraction of information for historical treatment effects. It is, therefore, critical to carefully consider the estimand from historical studies in a similar way shown here to determine the margin and control response (particularly if meta-analyses are used), so that the estimates from historical studies are aligned with what is to be estimated for a new study. In practice, this will be challenging as it will never be known what the intended estimand was for studies conducted before the estimand framework. We recommend all trialists use the estimand framework and clearly clarify the targeted estimands. As the estimand framework is adopted, the use of historical information from conducted studies can be completely aligned when a new study is designed since estimands can be aligned.

## Conclusions

We have demonstrated how estimands can be constructed in a non-inferiority trial setting using a primary estimand (where all IEs were handled using a hypothetical strategy) and an additional estimand (where all IEs were handled using a treatment policy strategy). Further, we have shown the applicability and the value of using multiple imputation methods for a binary outcome as an estimator for the proposed estimands to deal with IEs which may lead to missing data and intermittent missing data. We believe using estimands with aligned estimators improves the historical PP/mITT analyses for non-inferiority studies by providing clarity on exactly what is being estimated.

## Supplemental Material

sj-docx-1-ctj-10.1177_17407745231176773 – Supplemental material for Handling intercurrent events and missing data in non-inferiority trials using the estimand framework: A tuberculosis case study Supplemental material, sj-docx-1-ctj-10.1177_17407745231176773 for Handling intercurrent events and missing data in non-inferiority trials using the estimand framework: A tuberculosis case study by Sunita Rehal, Suzie Cro, Patrick PJ Phillips, Katherine Fielding and James R Carpenter in Clinical Trials
