# Supplementary material for: Handling intercurrent events and missing data in non-inferiority trials using the estimand framework: A tuberculosis case study
Source: Clin Trials. 2023 Jun 5;20(5):497–506. doi: 10.1177/17407745231176773 (PMC10504812; doi:10.1177/17407745231176773)
Supplement: sj-docx-1-ctj-10.1177_17407745231176773 – Supplemental material for Handling intercurrent events and missing data in non-inferiority trials using the estimand framework: A tuberculosis case study [file sj-docx-1-ctj-10.1177_17407745231176773.docx]

**Supplemental material for the manuscript: Handling intercurrent events and missing data in non-inferiority trials using the estimand framework:**

**A tuberculosis case study.**

Appendix A:

Multiple imputation by fully conditional specification (FCS or chained equations) imputes using a series of univariate conditional models, formed as a regression of each partially observed variable, on all the other variables. This avoids the need to formally specify a joint multivariate density(1) and is arguably more flexible as the conditional distributions do not need to rely on the assumption of normality. However, when we have a long follow-up, and the probabilities are close to 1 or 0 for a binary outcome, a modification to this procedure is required to avoid computational issues such as collinearity and/or perfect prediction. This issue is especially acute in TB studies such as REMoxTB, which has 17 visits, a binary outcome, and long sequences of positive (presence of TB) and negative (absence of TB) results at the beginning and end of a study. In such settings, an extension to FCS, *the two-fold approach*, a doubly iterative procedure should be used. Below, we introduce multiple imputation for a single outcome followed by details on the fully-conditional specification approach before outlining two-fold FCS.

*Logistic imputation for an outcome at a single timepoint:*

For binary outcomes the following logistic regression model is used for imputation

$logit\left( a \right)$ = $log\left( \frac{a}{1-a} \right)$ = $X\beta$

where *a* represents the probability of failure or success (in TB studies this is typically classed as a treatment failure/relapse). *β* is a vector of logistic regression parameters:

$$\beta= \left[ \begin{aligned} \beta_{1} \\ \beta_{2} \\ . \\ . \\ . \\ \beta_{j} \end{aligned} \right]$$

and *X* is a vector of observed covariates where $X_{i,j}$is the jth covariate for each patient $i$:

$$X= \left[ \begin{matrix} 1 & X_{1,1} & X_{1,2} & \cdots& X_{1,j} \\ 1 & X_{2,1} & X_{2,2} & \cdots& X_{2,j} \\ \vdots& \vdots& \vdots& \ddots& \vdots\\ 1 & X_{i,1} & X_{i,2} & \cdots& X_{i,j} \end{matrix} \right]$$

From this model, the posterior mean $\left( \hat{\beta} \right)$ and the variance-covariance matrix $\left( V\left( \hat{\beta} \right) \right)$ of $\beta$ is approximated by maximum likelihood estimation. The posterior distribution of *β_i_* assumes a multivariate normal distribution.

To impute one dataset(2):

1. Draw $\beta_{m}$ from N$\left( \left( \hat{\beta} \right), V\left( \hat{\beta} \right) \right).$
2. Impute from the inverse logit function using the drawn values of *β_m_* for each patients’ missing observation:

$${logit}^{-1}{(X}_{i}\beta_{m})= \frac{exp\left( X_{i}\beta_{m} \right)}{1+exp\left( X_{i}\beta_{m} \right)}$$

3. Independently for each patient $i$ with missing data draw a random number, $\left( R_{i} \right)$, from a uniform probability distribution, U(0,1).
   1. If $R_{i}$>${logit}^{-1}\left( X_{i}\beta_{m} \right)$ 0 is imputed for the missing observation, otherwise 1 is.

The process is iterated for $m=1,\ldots, M$ imputations. Each imputed data is then analysed using the analysis model of interest and results are combined using Rubin's Rules(2).

*Multiple imputation:*

Multiple imputation was first introduced by Rubin in 1978(2). The concept of multiple imputation is that missing data are imputed more than once based on the distribution of the observed data, including an element of randomness to reflect the uncertainty about the missing values(2). Multiple imputation assumes that the missing data are missing at random (MAR). That is, the probability of data being missing for variable Y is independent of the unobserved data (Y_miss_) conditional on the observed data (Y_obs_). Imputations are drawn from the joint posterior predictive distribution, f(Y_miss_|Y_obs_), to form one imputation set. This is repeated for as many imputations as required. Following imputation, results across imputed data sets are combined using Rubin’s rules, to provide one overall multiple imputation estimator and estimate of variance. In practise, with longitudinal missing data, imputations may be drawn using either a full joint multivariate modelling approach or by full conditional specification.

*Multiple imputation by fully conditional specification (FCS) to impute observations at more than one time point:*

When an outcome (Y) is imputed by treatment arm (X_trt_) which has no missing values, the multiple imputation model can be denoted by f(Y_mis,M_,Y_obs_,X_trt_)_M_ for *M* imputed data sets where Y_mis_ and Y_obs_ corresponds to missing and observed outcomes(3). To impute missing observations, let $\tilde{z}$ represent the imputed values. Then for one imputed dataset:

1. For a vector of unknown parameters, say $\hat{\phi}$, calculate the posterior distribution p($\hat{\phi}$|Y_obs_,X_trt_).
2. Draw a random parameter, $\hat{\phi}$^*^, from the multi-variate normal distribution: $\hat{\phi}$^*^~N($\hat{\phi}$,Var($\hat{\phi}$)).
3. Set $\hat{\phi}$ = $\hat{\phi}$^*^ and draw a value, $\tilde{z}$, from the conditional posterior distribution of p(Y_mis_ | Y_obs_,X_trt_, $\hat{\phi}$ = $\hat{\phi}$^*^).

The FCS algorithm proposed by van Bureen et al samples iteratively to account for any dependence on the estimated model parameters(4). For multivariate Y, which is incomplete, using FCS imputes the missing data one variable at a time. This is an iterative process cycling through all variables, possibly with different conditional specifications several times. For one cycle, the joint model multiple imputation is approximated by regressing the observed part of (Y_j_) on all other remaining variables, where for TB data *j* takes on a positive or negative culture result, and missing values take their current imputed values. Using the logistic regression model as specified above ($logit^{-1}(X_{i}\beta_{m})$) the missing values of Y_j_ are generated(3). Following this first cycle, the initial starting values are replaced by imputed values(5). A number of cycles are run and the imputations are taken from one final cycle through univariate models(1).

The use of only using FCS means that imputations are performed independently, and so any information gained from the current imputed observation is not considered at future time points. This has two implications i) imputing separately for each visit does not take into account correlations between observations per patient and ii) for several follow up visits the regression model used for imputation may be over-fitted(1).

Appendix B:

*Reference based sensitivity analyses*

*Outline of reference-based imputation for continuous data*

Consider a two-arm trial with a continuous outcome intended to be measured at a fixed number of scheduled follow-up times. Suppose a number of patients withdraw before the end of the follow-up, and their post-withdrawal data are missing. The following then occurs:

1. Assume MAR and fit — separately in each arm — an unstructured multivariate normal model to all the observed outcome data,
2. For each patient who withdraws — taking into account their reason for withdrawal — we build a joint multivariate normal distribution for the whole of their follow-up from (or by *reference* to) the parameters estimated in the first step,
3. Constructs from 2) the conditional distribution of each patient’s missing outcome data given their observed outcome data, and
4. we impute each patient’s missing outcome data from their distribution. Since the distribution in step two uses information at withdrawal, the resulting imputed values come from a missing not at random imputation model.

There are many options (jump to reference, copy increments in reference, copy reference, last mean carried forward and missing at random) for constructing the distribution in Step 2, and these are reviewed and detailed in Cro(6); we may choose a different option for each patient, but typically we choose a common option for each treatment arm.

Appendix C:

*Adaptive rounding algorithm*

To back-transform to binary data, the following adaptive rounding algorithm is used:

1. For binary variable *t* in imputed dataset *m=1, ..., M* let $\bar{Y}$_t,m_ be the mean of the observed and imputed values at time t.
2. The binomial distribution is approximated to the normal distribution(5).

Let φ(.) denote the cumulative distribution function of the standard normal. A threshold is constructed such that:

$C_{t,m}$= $\bar{Y}_{t,m}$ - $\varphi^{-1}$ ($\bar{Y}_{t,m}$) $\sqrt{\bar{Y}_{t,m}(1-\bar{Y}_{t.m})}$

1. Imputed values are re-coded as 0 if Y_t,m_≤C_t,m_ and 1 if Y_t,m_>C_t,m_.

Appendix D:

Example code of using *mimix* and calculation of the adaptive rounding algorithm in Stata:

******************************

* Use mimix *

******************************

/* 1. Jump to reference */

mimix t_fail alloc, id(trial_no) time(w_week) method(`v') refgroup(1) ///

clear m(50) seed(789562)

********************

* CREATE THRESHOLD *

********************

* A. Create mean observations at each window *

qui forvalues m = 0/50 {

qui forvalues i = 1/4 {

su t_fail if w_week == `i' & _mi_m == `m'

scalar y`i'_m`m' = r(mean)

* the inverse cumulative standard normal distribution: if normal(z)

* = p, then invnormal(p) = z

scalar phi`i'_m`m' = invnormal(y`i'_m`m')

* B. calculation of threshold //

scalar w`i'_m`m' = (y`i'_m`m'*(1-y`i'_m`m'))

/* THRESHOLD */

scalar C`i'_m`m' = (y`i'_m`m' - phi`i'_m`m'*(sqrt(w`i'_m`m')))

* c. recode values

// if the imputed value is <= than the threshold impute as 0

replace t_fail = 0 if t_fail<= C`i'_m`m' & t_fail!=0 & t_fail!=1 & ///

w_week == `i' & _mi_m==`m' & _mi_m!=0

// if the imputed value is > the threshold impute as 1

replace t_fail = 1 if t_fail> C`i'_m`m' & t_fail!=0 & t_fail!=1 & ///

w_week == `i' & _mi_m==`m' & _mi_m!=0

*******************************

* END OF TRESHOLD CALCULATION *

*******************************

//assert t_fail == 0 | t_fail == 1 if _mi_m!=0

}

}

**References**

1. Nevalainen J, Kenward MG, Virtanen SM. Missing values in longitudinal dietary data: a multiple imputation approach based on a fully conditional specification. Statistics in medicine. 2009;28(29):3657-69.

2. Rubin DB. Multiple Imputation for Nonresponse in Surveys. New York: John Wiley and Sons; 1987.

3. Welch CA, Petersen I, Bartlett JW, White IR, Marston L, Morris RW, et al. Evaluation of two-fold fully conditional specification multiple imputation for longitudinal electronic health record data. Statistics in medicine. 2014;33(21):3725-37.

4. van Buuren S, Boshuizen HC, Knook DL. Multiple imputation of missing blood pressure covariates in survival analysis. Statistics in medicine. 1999;18(6):681-94.

5. Carpenter JR, Kenward, M. G. Multiple Imputation and its Application: New York; 2013.

6. Cro S, Carpenter JR, Kenward MG. Information-anchored sensitivity analysis: theory and application. The Authors Journal of the Royal Statistical Society: Series A (Statistics in Society). 2019.
